# Production of Flocculants, Adsorbents, and Dispersants from Lignin

**DOI:** 10.3390/molecules23040868

**Published:** 2018-04-10

**Authors:** Jiachuan Chen, Armin Eraghi Kazzaz, Niloofar AlipoorMazandarani, Zahra Hosseinpour Feizi, Pedram Fatehi

**Affiliations:** 1 Key Laboratory of Pulp and Paper Science and Technology of Ministry of Education, Qilu University of Technology (Shandong Academy of Sciences), Jinan 250353, China; chenjc@qlu.edu.cn; 2 Chemical Engineering Department, Lakehead University, 955 Oliver Road, Thunder Bay, ON P7B 5E1, Canada; aeraghi@lakeheadu.ca (A.E.K.); nalipoor@lakeheadu.ca (N.A.); zhossein@lakeheadu.ca (Z.H.F.)

**Keywords:** lignin, flocculants, dispersants, chemical reaction, product analysis, biorefining

## Abstract

Currently, lignin is mainly produced in pulping processes, but it is considered as an under-utilized chemical since it is being mainly used as a fuel source. Lignin contains many hydroxyl groups that can participate in chemical reactions to produce value-added products. Flocculants, adsorbents, and dispersants have a wide range of applications in industry, but they are mainly oil-based chemicals and expensive. This paper reviews the pathways to produce water soluble lignin-based flocculants, adsorbents, and dispersants. It provides information on the recent progress in the possible use of these lignin-based flocculants, adsorbents, and dispersants. It also critically discusses the advantages and disadvantages of various approaches to produce such products. The challenges present in the production of lignin-based flocculants, adsorbents, and dispersants and possible scenarios to overcome these challenges for commercial use of these products in industry are discussed.

## 1. Introduction

Greenhouse gas emission is a major driver of global warming; mainly caused by human activities, such as fossil fuel combustion, deforestation, livestock farming, use of synthetic fertilizers, and industrial processes. Contamination of global freshwater systems with industrial chemicals is another crucial problem calling for the generation of nontoxic chemicals and transition to more sustainable processes [1,2]. Currently, many oil-based chemicals are used in industry for a variety of purposes, and unwillingly some will be released to the environment, causing wildlife and environmental issues, consequently. Also, these oil-based chemicals are heavily used in treating water and wastewater systems [3,4]. These commercial materials are mainly expensive, inefficient, and sometimes environmentally unfriendly, raising the need for being replaced by environmentally friendly analogs, such as biomass based value-added products.

Lignin is the most abundant natural aromatic polymer composing 18–35 wt. % of wood [5,6]. It was estimated that (5–36) × 10^8^ tons of lignin are produced annually [7]. In woody plants, lignin plays a critical role in providing strength to fiber walls, controlling fluid flow, and protecting against enzymatic degradation [8]. Lignin contains phenylpropanoid units originating from three aromatic alcohol precursors (monolignols), p-coumaryl, coniferyl, and sinapyl alcohols [9]. It was reported that 50 million tons of lignin are produced annually only by the pulping industry; however, the majority of lignin is burned and used as a fuel source, implying that lignin is currently under-utilized [9,10]. Lignin’s physicochemical advantages are classified as: (a) being rich in functional groups; (b) having high adsorption capacity; and (c) having high potential for value-added product production [10]. Interestingly, lignin’s renewable, nontoxic, and biodegradable nature allows for its effective valorization into value-added products [11]. The efforts to convert lignin derivatives into sensor components [12], biocomposites [13], biofuels [14,15], and hydrogels [16,17] are currently under development.

Lignosulfonate is a by-product of the sulfite pulping process, providing up to 90% of commercial lignin with an annual production of around 1.8 million tons [18,19,20,21,22,23]. The hydrophobicity, water solubility, and negative charge density of lignosulfonate, in addition to its thermal stability, renewability and eco-friendliness [24], make this polymer a promising candidate to be used in industry [21,24]. Kraft lignin is produced via pulping of wood chips using sodium sulfite and sodium hydroxide, while alkali lignin is produced via pulping of wood chips using sodium hydroxide [18,19,20,21,22,23]. Kraft lignin and alkali lignin are not water soluble at pH 7 in opposition to lignosulfonates [18,19,20,21,22,23].

Technical lignin’s application as a flocculant and dispersant has received a considerable attention [25], which is the focus of this review paper. In this work, the advantages and disadvantages of using lignin for producing flocculants, coagulants, and dispersants are also critically reviewed.

## 2. Flocculant

**Figure 1:**
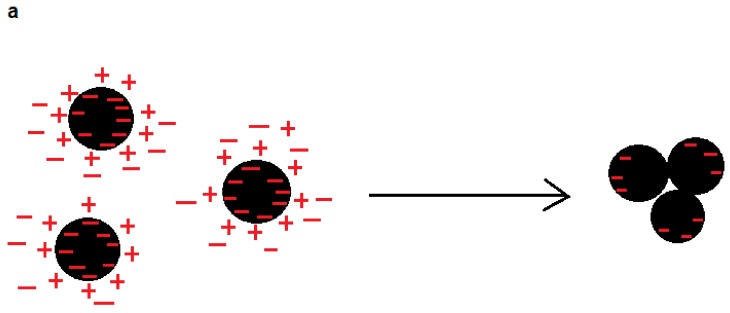

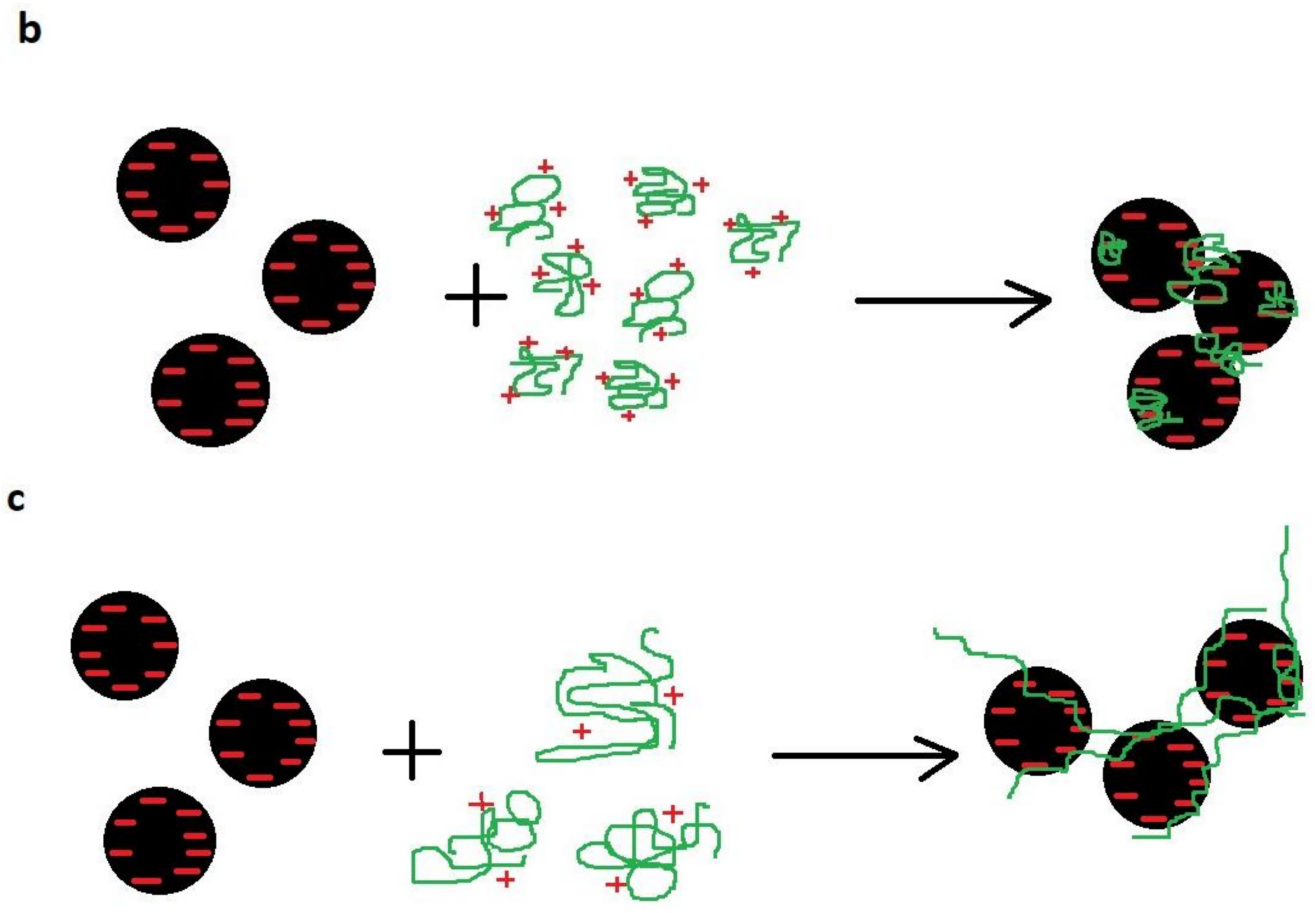
Mechanisms of flocculation: (**a**) charge neutralization; (**b**) patching; and (**c**) bridging.

Flocculants have been extensively used in treating wastewater effluents with the aim of removing colloidal particles from the solutions and suspensions [4,26]. Flocculants aggregate colloidal particles via charge neutralization, patching, and bridging mechanisms as shown in Figure 1. In the charge neutralization mechanism, the charges of colloidal particles are neutralized by adsorption of flocculants, and thus repulsion force existing between adjacent particles is diminished/reduced, which causes these particles to agglomerate via developing van der Waals interaction, for example (Figure 1a). In the patching mechanism, flocculants adsorb on a colloidal particle due to their opposite charges, and they neutralize a part of the particle’s surface. Due to their opposite charges, the adsorbed flocculants will act as anchoring points for another adjacent particle for aggregation (Figure 1b). In the bridging mechanism, high molecular weight flocculants adsorb on particles. Due to their large sizes, the flocculants bridge particles and hence generate large flocs (Figure 1c). Although synthetic organic flocculants have been mostly used in industry [27,28,29], they are non-biodegradable, inefficient, and expensive [30]. Biomass based flocculants can be produced to replace synthetic flocculants. Table 1 classifies different kinds of modified lignin used as flocculants in various fields [31]. In the following sections, the application of lignin-based flocculants in different wastewater treatment systems is described.

### 2.1. Flocculants for Wastewater Systems

**Figure 2:**
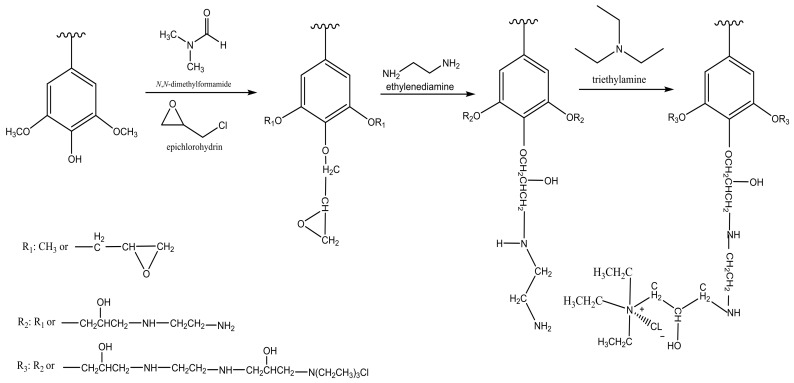
Reaction scheme for producing crosslinked amine-based sludge flocculant [34]. The sludge contained mainly lignin and hence the structure of lignin was selected to represent sludge as the raw material in this reaction.

To remove natural organic substances from wastewater, the most frequently used and economically feasible method is coagulation/flocculation [32]. Generally, hydrophobic and high molecular weight compounds could be removed via coagulation. However, this method is inefficient for removing low molecular weight and hydrophilic compounds from wastewater [33]. To address this problem, Li and coworkers [34] synthesized a sludge-based flocculant by grafting amine groups onto the organics of sludge (Figure 2). The sludge contained a major portion of lignin, and thus the chemical structure of lignin was considered for describing its reaction mechanisms with other chemicals in Figure 2. The reaction was conducted by reacting sludge with epichlorohydrin and *N*,*N*-dimethylformamide at a ratio of 1/1.6/1.6 (*w*/*v*/*v*) dried sludge/epichlorohydrin/*N*,*N*-dimethylformamide at 60–70 °C for 1 h. Then, ethylenediamine was added to the reactor at the ratio of 1/0.4 (*w*/*v*) dried sludge/ethylenediamine and the mixture was treated for another hour. Afterward, triethylamine was reacted with the product under the conditions of 1/1.2 (*w*/*v*) dried pulping sludge/triethylamine for two hours at 60–70 °C. The produced amine-crosslinked sludge-based cationic polymer used as a flocculant along with aluminum sulfate (AS) and poly aluminum chloride (PAC) for removing humic acid from a wastewater effluent. The AS and PAC were used for improving the efficiency of flocculation in a dual system. The results showed that the product could be used as a flocculant in this system and its efficiency was 25% higher under acidic conditions [34].

In another study, the sludge of a pulping process containing 45–50 wt. % lignin and 5–10 wt. % cellulose was used to produce acrylamide-based polymers in a free radical polymerization system under alkaline pH (Table 1) [39]. The product of this reaction was used as a flocculant along with polyaluminum chloride (PAC) to treat humic acid solution [40]. However, the poor solubility of the flocculants at a higher pH was cited as the main problem of this flocculation system.

Lignosulfonate can be used in the production of flocculants. Lignosulfonate has been reported to have the highest molecular weight among all lignin products [21]. Generally, it was suggested that the higher the molecular weight of the polymer, the better its flocculation performance [22]. The water solubility of lignosulfonate has led to its vast utilization as a flocculant in wastewater systems [21]. Table 2 shows lignosulfonate applications as a flocculant in various fields. The charge density and molecular weight of these samples were not reported in these reports.

Liu and coworkers grafted olefin monomers to lignosulfonate by using radiation graft polymerization and used the resulting natural-based polymers as flocculants for removing furfural from wastewater [42]. In another work, lignosulfonate was used as a flocculant along with a high molecular weight linear polyacrylamide-based polymer for flocculating particles in a mining waste. The dual polymer system having lignosulfonate and polyacrylamide-based polymer at the dosages of 1 and 2.5 g/L, respectively, was found to be effective as it increased the chord length of the particles from 20 to 145 µm for the flocs, while decreasing the number of particle in suspension from 100,000 to 80,000 as determined by focused beam reflectance measurement (FBRM) analysis [43].

Even though lignosulfonate is water soluble [22], there are some barriers in its use: (1) it is less available than kraft lignin worldwide since the kraft pulping process is more common than the sulfite pulping process; (2) its high sulfur content is a limitation for some applications such as polymer synthesis; (3) sulfur removal from lignosulfonate is challenging since it is chemically bonded to lignin; and (4) the sulfite spent liquor containing lignosulfonate contains hemicelluloses, indicating the impurity in the produced lignosulfonate [45].

### 2.2. Flocculants for Dye Removal

Textile effluents contain a significant amount of dyes, which are generally non-biodegradable and have a high level of toxicity and strong color [38]. To treat this source of environmental pollution [46], adsorption, oxidation, hyper-filtration, biodegradation, flocculation, and ozonation processes were considered in the past [47,48,49,50,51,52]. Inorganic coagulants including aluminum sulfate, ferric chloride, and polyaluminum salts are used as flocculants for dye removal due to their low costs; however, these inorganic coagulants need to be applied in high concentrations, resulting in a large volume of sludge. The use of high concentration of ferric or aluminum ions generally yields a high concentration of salts in the solution after treatment, which has a harmful effect on the ecosystem [38]. 

Lignin has been aminated (Table 1) for removing anionic dyes from solutions. In a work conducted by Fang and coworkers, a quaternary ammonium salt was grafted onto kraft lignin with a mass ratio of 2/1 of trimethyl quaternary ammonium/lignin at 70 °C [35]. The modified lignin at 2–3 g/L dosage showed 94.02% removal of Acid Black dye from a 0.1 g/L dye concentration in acidic conditions (pH 2–3) [35]. It was also discovered that the aminated lignin could remove 94.91% of Acid Black ATT (0.1 g/L concentration) and 97.11% of Methylene Blue (0.1 g/L concentration) at 30 °C and pH 3 and 8, respectively [36]. Kong et al. (2015) grafted glycidyl-trimethylammonium chloride (GTMAC) onto softwood kraft lignin (Table 1) in the molar ratio of 2/1 GTMAC/lignin for 1 h at 70 °C, in which aminated lignin with the charge density of 1.10 meq/g was generated [11]. In this reaction, GTMAC reacts with the phenolic group of lignin under alkaline conditions and renders lignin cationically charged and water soluble. The use of this lignin as a flocculant for the removal of Remazol Brilliant Violet (V5), Reactive Black 5 (B5), and Direct Yellow 50 (Y50) (100 mg/L concentration) led to more than 87% dye removal at the lignin concentrations of 95, 235, and 375 mg/L for V5, B5, and Y50, respectively [11]. In another study, enzymatically hydrolyzed cornstalk lignin was polymerized via the Mannich reaction with dimethylamine, acetone, and formaldehyde. The addition of 75, 50, and 35 mg/L of this cationic flocculant in Acid Black, Reactive Red, and Direct Red dyes solutions (100 mg/L) led to 97.1, 98.3, and 99.5% removals, respectively [11]. He and coworkers reported that a ternary graft copolymer of lignosulfonate-acrylamide-chitosan could be used as a flocculant in wastewater treatment systems. The copolymer was produced with a chitosan/sodium lignosulfonate/acrylamide mass ratio of 1.5/1.5/3 mixed with potassium persulfate/sodium thiosulfate in the mass ratio of 0.05/0.05 and reacted for 5 h at 60 °C. Three different dyes of Reactive Black 5 (Rb-5) (neutral), Methyl Orange (MO) (cationic), and Acid Blue 113 (Ab-113) (anionic) were used to simulate textile wastewater and the produced amphoteric copolymer was used as a flocculant in the system. The polymer removed 50, 85, and 98% of dyes (50 mg/L concentration) when 335, 135, and 35 mg/L of the polymer were used for MO, Rb-5, and Ab-113, respectively. The flocculation mechanism was determined to be charge neutralization in this work [44].

Lignin has also been modified to increase its anionic charge density for removing cationic dyes from solutions. In one study, softwood kraft lignin was oxidized (Table 1) with nitric acid (20 wt. % based on lignin, 60–100 °C for 0.5–4 h) and then sulfomethylated under the conditions of 1/1 mol/mol formaldehyde/lignin, 0.5/1 sodium metabisulfite/lignin, and 4 wt. % sodium hydroxide concentration at 60–100 °C for 1 h. The produced lignin had a charge density of −4.61 meq/g and the molecular weight of 18,300 g/mol, and was able to coagulate 99.1% of ethyl violet dye (200 mg/L concentration) at a 300 mg/L dosage [41]. Couch et al. (2016) reported that, thermomechanical lignin-carbohydrate complex (LCC), containing 76 wt. % lignin and 24 wt. % of hemicellulose, was oxidized (Table 1) with nitric acid under different concentrations of 70 and 30 wt. % at 60–100 °C for different times (20–120 min). The oxidized lignin had a charge density of −3.02 meq/g and molecular weight of 6270 g/mol. The use of this product as a flocculant resulted in 70–80 wt. % of Ethyl Violet and 80–95% of Basic Blue dye removals from simulated solutions [37]. Generally, the charge density of this modified lignin had a significant impact on their performance since the main driving force for the removal was the charge neutralization mechanism [53].

Effluents of the textile industry may contain salts and have different pH. It was reported that the reduction of pH improved the efficiency of aminated lignin in dye removal. The presence of salts (NaCl and NaNO_3_) up to 0.05 mol/L concentration did not affect their performance significantly [38]. However, NaCl (at a 3 g/L concentration) reduces the efficiency of oxidized lignin in Basic Blue dye removal from 84% to 77% [37]. 

However, the main barrier to produce lignin-based flocculants are the complex and heterogeneous structure of the flocculants [54,55]. Also, lignin obtained from different processes (sulfite, kraft, and organosolv) have different physical and chemical characteristics, thereby affecting their modification processes and product performance [56]. The current challenge in producing lignin-based flocculants is to identify environmentally friendly and cost-effective processes for lignin modification (Table 1) in order to induce flocculants with a high charge density, molecular weight, and hydrophilicity. On the other hand, lignin contains many functional groups such as phenol hydroxyl, methoxy, hydroxy, and carboxy groups [57,58], which makes it feasible to engraft the functional groups to its structure [59].

## 3. Adsorbent

### 3.1. Adsorbent for Heavy Metals

Wastewater from mining operations, fossil fuel combustion, metal plating, and tanneries contain heavy metals and poses environmental concerns [60]. Application of lignin to adsorb these heavy metals from solutions has shown a profound efficiency [61]. A summary of lignin application in metal removal is compiled in Table 3. 

**Figure 3:**
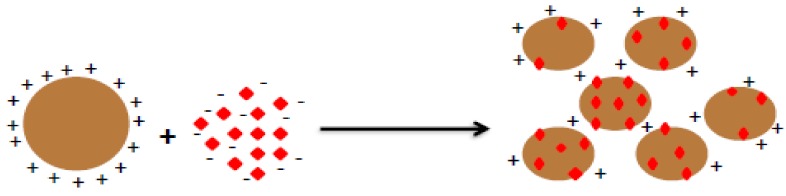
Mechanism of adsorbents in adsorbing ions.

Generally, adsorbents have charges and these charges will interact with ions in wastewater via the charge neutralization mechanism. The results of this interaction are the complexation of ions with the adsorbents, as shown in Figure 3. The produced complexes may be removed from wastewater effluents via filtration and/or sedimentation.

Kraft lignin, without modification, has been used as an adsorbent in many simulated wastewater systems. In one report, bonding strength among various ions and kraft lignin was reported to be in the order of Pb(II) > Cu(II) > Cd(II) > Zn(II) > Ni(II) [62]. Results demonstrated the higher efficiency of lignin’s phenolate group than lignin’s carboxylate groups in coagulating metals [62]. In another study, the adsorption of heavy metals on kraft lignin followed the order of Cr(VI) > Cd(II) > Cu(II) > Zn(II) [66]. As shown in Table 3, lignin had different efficiencies in metal removal as differently sourced lignin contained varied properties originating from wood species and pulping process [62,63,64,65,66,67,68].

**Figure 4:**
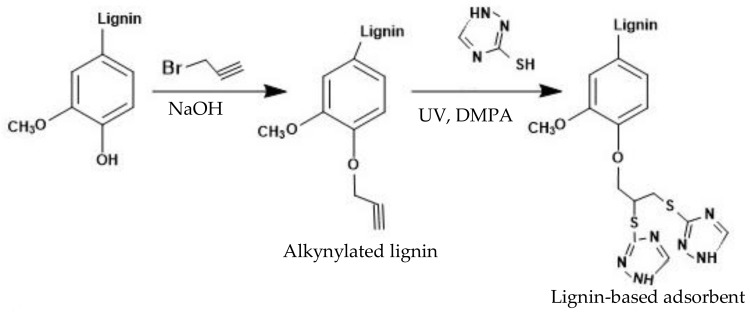
Schematic reaction mechanism of thiol-yne alkynylation lignin [69].

Modification of lignin has also been examined as a means to produce adsorbents for heavy metal removal. One study introduced secondary amino groups on enzymatic hydrolysis lignin’s backbone via reacting with poly(*N*-methylaniline, PNMA) for 4 h at 25 °C [13]. Introduction of methyl and imino groups onto lignin enhanced electron donating properties of the final product. The product removed silver ions from wastewater. A high silver uptake of 1556.8 mg/g was reported for this modified lignin. In the past, influence of aminated-Fe lignin complex on ${\;\mathrm{PO}}_{4}^{3-}$ uptake from wastewater was studied [71]. First, lignin was aminated by triethylenetetramine in an alkaline solution for 4 h at 65 °C. Then, aminated lignin was chelated with Fe${\mathrm{Cl}}_{3}$ by stirring for 4 h at 35 °C to allow the uptake of ${\mathrm{Fe}}^{3+}$. When the modified lignin was applied to wastewater system, the phosphate-Fe-lignin complexes were formed with the particle size of 452.4 nm. Results reported a maximum ${\mathrm{PO}}_{4}^{3-}$ removal capacity of 90%. It was confirmed that Fe(III) was the active site for adsorption, and charge interaction was the main interaction force between phosphate and iron. Another novel modification for alkynylated lignin production was performed through thiol-yne reaction to remove different ions from water [69], as shown in Figure 4. In the first reaction, alkynylated lignin was prepared via reacting lignin and propargyl bromide under alkaline conditions for 2 h and 75 °C (Figure 4). In the next reaction, thiol-yne click reaction was conducted on alkynylated lignin, in which 2,2-dimethoxy-2-phenylacetophenone (DMPA) and 1,2,4-triazole-3-thiol monomers were reacted with the modified lignin in tetrahydrofuran under UV radiation. Introduction of thio-triazole units into lignin led to an increased molecular weight of the product (6756 g/mol) with an alkynylation degree of 2.47 mmol/g. The produced coagulant exhibited a selective adsorption capacity in the order of Cd(II) > Pb(II) > Cu(II) > Ni(II) > Zn(II), which is consistent with what was reported for unmodified lignin [66]. 

In all cases stated above on modified lignin, maximum adsorbed mass is correlated to the charged groups and molecular weight of lignin, which in turn enhanced the solubility of modified lignin. But even insoluble lignin seems to be an efficient adsorbent. In one study, a two-step process to synthesize porous lignin has been developed [70]. Mannich reaction to graft polyethylenimine (PEI) onto alkali lignin matrix at 90 °C for 5 h was first implemented to introduce amine groups to lignin. Then, carbon disulfide was used for completing modification, while introducing dithiocarbamate groups to lignin. The final product possessed 8.5 mmol/g of nitrogen and 2.8 mmol/g of dithiocarbamate groups. The higher surface area of this adsorbent (22.3 $m^{2}$/g) compared to lignin (1.8 $\;m^{2}$/g) confirmed the porous structure of the functionalized lignin matrix. The final product exhibited excellent adsorption performance toward lead ions with 90% removal from the solution (120 mg/g). However, the two stages of the Mannich and esterification reaction may be too complex to be implemented in industry. Yang and coworkers formed a lignin-based interconnected foam in an oil/water interface by in-situ polymerization containing lignin (1 wt. %) and melamine formaldehyde (25 wt. %) in a water/toluene mixture at 60 °C for 4 h [73]. The product was employed to remove ${\mathrm{Cu}}^{2+}$ and ${\mathrm{Cd}}^{2+}$ from wastewater. The highest adsorbed capacities of 73.2 mg/g and 142.3 mg/g were reported for ${\mathrm{Cu}}^{2+}$ and ${\mathrm{Cd}}^{2+}$, respectively. 

### 3.2. Adsorbent for Dyes 

Table 4 demonstrates that lignocellulosic material can be a good candidate to remove dyes from aqueous media. Unmodified wheat straw lignin with a molecular weight of 3510 g/mol and carboxylate content of 3.8 mmol/g was applied for adsorbing Brilliant Red dye [50]. Thermodynamic parameters confirmed a spontaneous endothermic adsorption process. The driving force for adsorption stems from electrostatic interaction between the oppositely charged groups of lignin and dye. The highest adsorption capacity was 10.13 mg/g at 20 °C. 

The counterions attached to lignin were proposed to influence its adsorption efficiency. In one work, carboxymethylated lignin was produced under basic conditions via reacting with monochloroacetic acid [75]. The product was treated with ${\mathrm{FeCl}}_{3}\cdot 6H_{2}O$ solution for 24 h to ensure that ${\mathrm{Fe}}^{3+}$ was coupled to the lignin backbone. The final product with a molecular weight of 1890 g/mol was shown to be a potential adsorbent for Brilliant Red dye. The high adsorbed amount of 73.6 mg/g indicated an electrostatic attraction between carboxymethyl Fe-based lignin and an anionic dye. In another study, ${\mathrm{Al}}^{3+}$ and ${\mathrm{Mn}}^{2+}$ based carboxymethylated lignins were produced and the coagulation of Procion blue dye with these lignins was studied [77]. Al-lignin and Fe-lignin complexes showed similar adsorption capacity [75,77]; while the Mn-based lignin complex yielded a lower adsorption capacity. This difference may be attributed to the ion charge capacities of the metals. 

Li and coworkers used larch-based and poplar-based lignins to form esterified lignin via reacting lignin with maleic anhydride in acetone at 60 °C [78]. The esterified lignins, along with ${\mathrm{Fe}}_{3}O_{4}$ nanoparticles, were dissolved in tetrahydrofuran. After investigating the adsorption capacity of modified lignins for Methylene Blue dye, it was observed that larch-based adsorbent with the molecular weight of 3200 g/mol exhibited an adsorption capacity of 31.23 mg/g. Monolayer surface coverage of lignin with dyes was claimed to occur via van der Waals interaction between the benzene ring of the dye and the esterified lignin. Results obtained from this insoluble lignin suggests that adsorbent’s particle diameter and surface area play significant roles on its adsorption capacity. Also, the incorporation of ${\mathrm{Fe}}_{3}O_{4}$ may hinder the commercialization step to produce such adsorbent.

### 3.3. Adsorbent for Other Applications

Lignin appears to be an excellent adsorbent in chemical processes, which is tabulated in Table 5. As a 2,4,6-Trinitrotoluene (TNT) adsorbent for military wastes, the chlorinated/aminated lignin was prepared by reacting lignin with 1,2-dichloroethane monomer and aluminum chloride [79]. The chlorinated lignin was aminated via reacting with *N*,*N*-dimethylformamide and ethylenediamine monomers. Adsorption of TNT on lignin reached 55.7 mg/g, mostly through hydrogen bonding. 

In the past, lignin had also been used as an adsorbent to recover gold from the mining industry. In one report, kraft lignin in an acidic HCl solution was used to uptake gold at 40 °C [80], where 100% coagulation of gold and lignin was achieved due to the reduction of Au(III) ions by acidic ${\mathrm{Cl}}^{-}$ ions in a redox reaction. Thermodynamics of adsorption documented that the gold flakes were formed in an endothermic (ΔH >0) entropy-driven process (ΔS >0). This high removal of gold was attributed to attachment of Au${\mathrm{Cl}}_{4}^{-}$ complex to the positively charged surface of adsorbent through electrostatic interactions. However, high dissolution of lignin at elevated temperatures was a barrier for this process, and the use of other water insoluble lignin in developing adsorbents needs to be evaluated in the future. 

## 4. Dispersants

Dispersants are widely used for suspending colloidal particles in cosmetics, paints, pharmaceuticals, drilling mud, cement, and ceramic applications [87]. Many synthetic polymers have been used as dispersants [88,89,90]. However, their toxic nature and/or non-biodegradability limits their use in industry [89]. The semi-natural or natural polymers have been proposed to be utilized as dispersants [91]. Lignin has been modified and used as a dispersant for different applications, as listed in Table 6.

**Figure 5:**
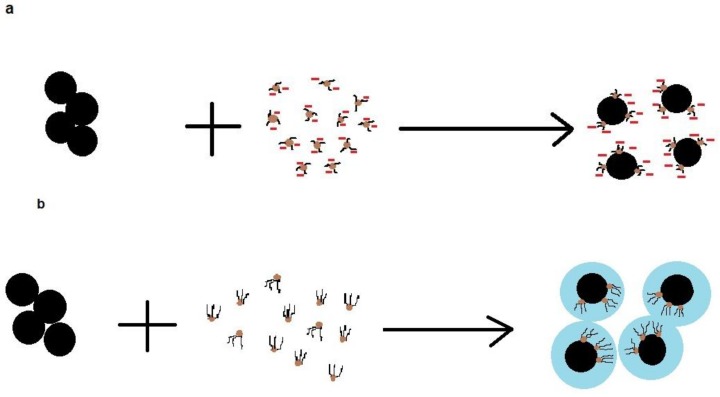
Mechanism of dispersants in stabilizing particles in suspensions (**a**) surface charge density effect and (**b**) hydrophilicity effect.

Figure 5 shows the mechanism of dispersants in stabilizing particles in suspensions. In Figure 5a, the adsorption of a dispersant, e.g., lignin, increases the surface charge density of particles, which enhances repulsion force development between particles and stabilizes them in suspensions. In Figure 5b, the adsorption of a dispersant, e.g., lignin, improves the hydrophilicity of particles and thus facilitates the interaction of water molecules and hydrated particles, thus reducing the hydrophobic/hydrophobic interaction developed between particles to prevent their agglomeration [92,93]. 

### 4.1. Dispersant for Dyes

Dispersive dyes have very low solubility in aqueous solutions. Adding dispersants is beneficial for preventing agglomeration of dye particles and for stabilizing dye dispersion [103]. Dispersants prevent the agglomeration of particles by introducing steric hindrance and electrostatic force among particles [109,110]. Commercial dispersants are mainly naphthalene-sulfonated formaldehyde and acid-phenol-formaldehyde condensates. Sulfonated lignin has been used as a dispersant for dye dispersion.

Qin and coworkers produced a dispersant for dye suspension through hydroxypropyl sulfonation of pinewood alkali lignin (HSL) (Table 6) under the conditions of 10/3.5 (*w*/*w*) lignin/sodium 3-chloro-2-hydroxy-propanesulfonate at 90 °C for 2 h, which was followed by crosslinking reaction with epichlorohydrin for 1 h [103]. The produced products had a molecular weight of 11,020 g/mol and contained 2.10 mmol/g of sulfonate group. The produced polymers and commercial sodium naphthalene sulfonic acid formaldehyde (SNF) dispersant showed similar results in dispersing dye particles.

It was observed that temperature affected the dispersion process in that a more dispersed dye solution was observed at a lower temperature of 25 °C [103]. The molecular weight of the sulfonated product also impacted the dispersion of dyes. The increase in the molecular weight of lignin-based dispersants from 8750 g/mol to 11,020 g/mol reduced the particle size of azo dye by 40%. On the other hand, increasing the molecular weight from 11,020 to 14,830 g/mol increased the particle size of an azo dye by 36% [103]. Therefore, the molecular weight of the dispersant should be tailored for achieving an acceptable efficiency.

### 4.2. Dispersant for Cement

Dispersants are used in the construction industry to boost the fluidity of concrete and decrease the water content of cement pastes [111,112]. Generally, sulfonated products are used as dispersants for cement admixtures. This is because sulfonate anions have more hydrophilic affinity than carboxylate anions [110]. However, common dispersants used in cement admixtures, such as sulfonated melamin-formaldehyde and sulfonated naphthalene-formaldehyde, have the disadvantages of enhanced concrete shrinkage, meager dispersibility, and pollution [113,114].

In the past, hardwood kraft lignin was sulfomethylated (Table 6) under the conditions of sodium hydroxymethylsulfonate/lignin ratios of 0.3–1.2 mol/mol in the temperature range between 80 and 140 °C and different periods of time (1–4 h). This process improved the anionic charge density and molecular weight of lignin to −1.60 meq/g and 53,360 g/mol, respectively. The application of this lignin (1.2 wt. %) improved the cement fluidity by 50% [94]. In another work, the oxidation (with 10–30 wt. % nitric acid, 60–100 °C and 0.5–4 h) and sulfomethylation (CH_2_O/lignin of 0.4–1.2 mol/mol and Na_2_S_2_O_5_/lignin of 0.3–0.7 mol/mol, 60–100 °C and 0.5–4 h) of softwood kraft lignin generated a sulfomethylated lignin with the charge density and molecular weight of −3.8 meq/g and 18,299 g/mol, respectively. The 0.5 wt. % dosage of this product increased the fluidity of cement from 65 mm to 200 mm [44].

In another study, kraft lignin from wheat straw was sulfonated (Table 6) by sodium sulfite in water in the temperature range between 45 and 55 °C. The product was hydroxymethylated via reacting with formaldehyde aqueous solution of 37% concentration at 98 °C for 3 h, which generated a sulfonated lignin with sulfonate group contents in a range between 1.5 to 3.7 mmol/g and the molecular weight of 30,000 g/mol. The results depicted that the use of this product yielded a fluidity of 290 mm, which was higher than that of a commercial naphthalene dispersant (250 mm) for a cement paste [101]. In another study, esparto grass lignin (Table 6) was sulfonated with sodium sulfite and formaldehyde, which generated sulfonated lignin with a molecular weight of 10,000 g/mol, and the sulfonate degree of 0.8 mmol/g. The use of sulfonated lignin in cement at the dosage of 0.4–0.6% showed a remarkable decrease in water requirement of cement (7–12%) [107]. In another work, a more complicated scenario was followed for producing sulfonated lignin [102]. At first, wheat straw alkali lignin was oxidized under the conditions of 10/2/0.1 (*w*/*w*/*w*) alkali lignin/H_2_O_2_/FeSO_4_ at 55–95 °C for 1 h. The product was then hydroxymethylated with 37% HCHO at 75 °C for 2 h. Finally, the sulfonation reaction was conducted with Na_2_SO_3_/lignin ratio of 1–2/4 (*w/w*) at 75–95 °C for 3 h. As reported, the produced sulfonated lignin could improve the dispersion of the cement admixture by 3% more than the commercial lignosulfonate [102]. The charge density and molecular weight seemed to impact the performance of sulfonated lignin in dispersing cement. 

Ozone was also used as an oxidizing agent for producing lignin-based dispersants. In one study, alkali lignin was ozonated under the conditions of 50–55 °C for 2 h (Table 6) and used as a dispersant for clay, titanium dioxide, cement and calcium carbonate suspensions (the suspension concentrations were 41, 35, 68.5, and 50 wt. %, respectively, at the dispersant concentrations of 0.126, 0.25, 1, and 0.25 wt. %, respectively). It was observed that the produced modified lignin could decrease the viscosity of clay, titanium dioxide, cement, and calcium carbonate suspensions up to 77, 98, 86, and 90%, respectively [95].

Table 7 lists the applications of lignosulfonates in industries as dispersants. The use of lignosulfonate as a dispersant for cement had been studied recently due to its acceptable performance and cost-effectiveness [115,116,117]. In some studies, lignosulfonate was modified to improve its dispersant performance in cement. In one study, calcium lignosulfonate was modified through oxidation (using hydrogen peroxide/lignosulfonate ratio of 0.12 (*w*/*w*) at 80 °C for 1.5 h), hydroxymethylation (using formaldehyde/lignosulfonate ratio of 0.35 (*w*/*w*) for 2 h at 80 °C) and sulfomethylation (using sodium sulfite/lignosulfonate mass ratio of 0.2 and formaldehyde/lignosulfonate mass ratio of 0.2 for 2 h at 90 °C). The performance of the product was evaluated as a dispersant for cement admixture. The adsorption of the oxidized, hydroxymethylated, and sulfomethylated lignosulfonates onto the cement surface was increased 7.5, 6.9, and 1.2 times with respect to that of the unmodified lignosulfonate, respectively. Although the adsorption of hydroxymethylated lignosulfonate was remarkable, its dispersion performance was rather poor compared to the oxidized and sulfomethylated lignosulfonates. The better dispersion performance of oxidized and sulfomethylated lignosulfonates was attributed to their higher negative charge densities [118]. In another work, sodium lignosulfonate underwent two consecutive reactions of oxidation (using 30% polyacrylic acid and 0.5% iron (II) sulfate for 2 h at 80 °C) and sulfomethylation (using 20% of formaldehyde and 30% of sodium sulfite for 3 h at 95 °C). These modifications led to an increase in both molecular weight (up to 10 times) and sulfonate content (up to two times) of the sodium lignosulfonate, which made the polymer 15% more efficient than the unmodified sodium lignosulfonate in enhancing the fluidity of a cement paste [115]. 

The counter ions attached to lignosulfonate may impact its efficiency as a dispersant. Among calcium, magnesium, sodium, and potassium lignosulfonates; calcium lignosulfonate was reported to reduce the cement viscosity, while magnesium lignosulfonate could hamper the cement admixture’s viscosity [117]. In addition, calcium lignosulfonate demonstrated the highest (11.1%) and sodium lignosulfonate showed the lowest (6.5%) capacity in reducing the water use of cement paste [117].

### 4.3. Dispersant for Mineral Particles 

Kaolin suspensions are used as raw materials for paper, ceramic, healthcare formulations, and pharmaceutical applications [129,130,131]. In these applications, the fluidity of a kaolin paste without settling is important to produce the products with appropriate properties [125,132]. By using dispersants, they adsorb on particle’s surfaces and change the overall surface charge density of the suspensions by inducing electrostatic or steric repulsion between particles. Various synthetic polymers, such as sodium tripolyphosphate and sodium polyphosphate, are used for dispersing kaolin suspensions, but their toxicity, price, and non-biodegradability are the main barriers for their implementation in industry [89,133].

**Figure 6:**
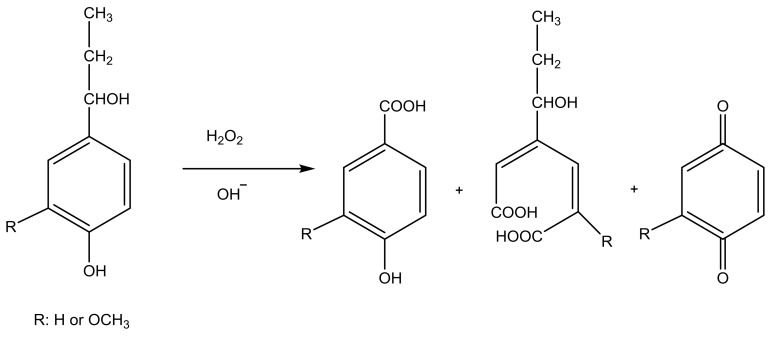
Oxidation of softwood kraft lignin [96].

Lignin based dispersants were also used for kaolin dispersion. He and coworkers oxidized softwood kraft lignin (Table 6) using hydrogen peroxide under varied reaction times (1–3 h) and temperatures (60–100 °C), which generated oxidized lignin derivatives with the charge density of −2.2 meq/g and molecular weight of 14,825 g/mol (Figure 6) [96]. Several lignin derivatives can be generated in this reaction, but the exact product of this reaction was not identified [96]. The anionic product improved the dispersion of kaolin suspensions by 18%. In another study, hardwood lignin (Table 6) was oxidized by using nitric acid (4–12 wt. % nitric acid/lignin at 100 °C for 1 h), and the product with the charge density of −3.6 meq/g and molecular weight of 30,243 g/mol showed the best performance in dispersing kaolin suspensions. Dispersion studies in this work suggested that both molecular weight and charge density had great impacts on the stability of kaolin particles [97].

Various lignin-based chemicals have been used to other suspensions, such as titanium dioxide slurry and graphite suspensions [98,100,106]. Graphite is extensively used in different industries, such as ceramics, and conductive coating, due to its corrosion resistance, superior electric, thermal conductivity, and chemical stability. Since graphite is a non-polar mineral with strong hydrophobicity, it is difficult to disperse it in polar solvents, such as water. However, in most of the applications, graphite particles should be dispersed in aqueous suspensions [134]. To address this challenge, wheat straw alkali lignin was carboxymethylated using monochloroacetic acid under the conditions of monochloroacetic acid/lignin ratio of 6/10 (*w/w*) at 70 °C for 90 min. As reported, carboxymethylated lignin with the dosage of 1 wt. % enhanced the suspension stability of graphite by around 4% [98].

Titanium dioxide (TiO_2_) is a material broadly used in plastics, inks, papermaking, paints, ceramics, and fibers, where its dispersion is critical [135,136]. However, titanium dioxide particles tend to agglomerate due to their large surface area. In order to address this problem, sulfomethylated wheat straw alkali lignin was modified by the horseradish peroxidase (HRP) under the conditions of ratio of 3/0.41 (*w/w*) lignin/formaldehyde at 95 °C for 1 h. After the reaction, the sulfonate and carboxylate groups, and the molecular weight of lignin were increased by 55%, 75%, and 470%, respectively. The dispersion performance of the produced polymer in titanium dioxide suspension showed a reduction of 84% in TiO_2_ particle size [106].

### 4.4. Dispersant for Coal-Water Slurry

Coal-water slurry is one of the alternative energy sources, which is cost-effective and easy to handle. Since this slurry contains a high coal content to meet the energy requirement, the water content of the slurry is of great importance. To reduce the water requirement, dispersants are used for diminishing the interaction among the particles in the slurry [133,137]. Lignosulfonate by itself may not be an effective dispersant for the coal-water slurry. As an inexpensive product, several studies were carried out to improve the efficiency of lignosulfonate as a dispersant for the slurry [138]. In one study, the molecular weight of lignosulfonate has been increased through the reaction of alkyl chain (conducted under alkaline condition using 1,6-dibromohexane in various mass ratios of lignosulfonate/1,6-dibromohexane of 1/0.08, 1/0.12, 1/0.16, 1/0.24, and 1/0.30, and potassium iodide for 8 h at 70 °C) and the products were used as dispersants for the coal-water slurry. It was disclosed that the lignosulfonate’s molecular weight was increased from 42,800 g/mol to 125,000 g/mol. All produced lignosulfonates worked better than the unmodified lignosulfonate and naphthalene sulfonate formaldehyde in reducing the coal-water slurry’s viscosity. This result was attributed to the higher molecular weights of the produced polymers, which may affect their adsorption affinity on the coal particles [128]. 

Ultrafiltration was used as a method to generate lignosulfonates with different molecular weights to refine them as dispersants for coal-water slurry [121,138]. The results showed that sulfonate group content of lignosulfonates with the lowest (2000 g/mol) and highest (17,000 g/mol) molecular weights were 1.36 and 1 mmol/g, respectively. Also, the carboxylate group content was determined to be 1.72 and 1.04 mmol/g for the highest and the lowest molecular weight lignosulfonate, respectively [138]. Overall, the molecular weight range of 10,000–30,000 g/mol was reported to be optimum for lignosulfonate to act as a dispersant for coal-water slurry [138]. 

### 4.5. Dispersant for Carbon Nanotubes Suspensions

Aqueous carbon nanotube (CNT) nanofluids have widely been studied for the preparation of nanocomposite materials with enhanced properties [139,140], such as large surface area, small size, remarkable electrical conductivity, and high mechanical strength [141,142,143]. However, they have poor solubility in water due to their tendency to agglomerate through van der Waals forces [144], which hampers CNTs’ utilization and application as dispersants. Although chemical and physical treatments have been suggested to address this problem [145,146], the disadvantage of chemical modification of CNTs is the disconnection of π-networks within CNTs, causing a decline in their electrical and mechanical properties [146,147]. Adding surfactants or polymers help cover CNTs by noncovalent interactions [148,149]. By adsorbing polar polymers on CNTs, intermolecular hydrogen bonding functionalized CNTs would enable CNT dispersion [150,151]. The most commonly used surfactants for this purpose are sodium dodecylsulfate, sodium dodecylbenzene sulfonate, octyl phenol ethoxylate, and hemadecyltrimethylammonium bromide [152,153,154,155,156,157]. 

Kraft lignin has been shown to have an efficient dispersion performance for CNTs. In one study, lignin (Table 6) was used without any modification as a dispersant for CNT, and its performance was compared with that of sodium dodecylbenzene sulfonate (SDBS). The results revealed that lignin (2 wt. %) could reduce the suspension’s viscosity by 70% (comparing to SDBS) without increasing the thermal conductivity of the suspension. Thus, it was suggested that lignin acted better than SDBS in dispersing the carbon nanotubes suspended in water at 0.55 vol. % [97]. 

Rochez and coworkers used spruce alkali lignin (Table 6) with the molecular weight of 14,000 g/mol to disperse multiwalled carbon nanotubes (MWCNT). By adding 10 g/L of lignin to 1 wt. % of MWCNT suspension, the transmission electron microscopy analysis showed that the nanotubes were distributed in the polymer matrix without morphological degradation or aggregation [104]. In another study, spruce wood alkali lignin, with the molecular weight of 14,000 g/mol (Table 6) was used to disperse multiwalled carbon nanotubes (MWCNT). Lignin was added to MWCNT with a lignin/MWCNT ratio of 1/6 (*w*/*w*). The lignin-MWCNT dispersion stability was monitored for several months at ambient conditions through which the lignin efficiency to disperse multiwalled carbon nanotubes was proved [104]. 

### 4.6. Dispersants for Other Suspensions

Dimethomorph, named (E,Z)-4-[3-(4-chlorophenyl)-3-(3,4-dimethoxyphenyl) acryloyl] morpholine, is a fungicide, which acts as a pesticide by protecting vegetables and plants from downy mildews, late blights, crown, and root rots [123]. The utilization of this pesticide requires its mixing with water. System stability and dispersion are critically important since the ingredients need to spread evenly on the plant surface after spraying [123]. Lin and coworkers dispersed a dimethomorph suspension by a synthesized pine kraft lignin-based polyoxyethylene ether (KL-PEG) [100]. KL-PEG production was carried out via reacting kraft lignin with poly(ethylene glycol) in a weight ratio of 100/0.8. The ethoxyethane-trifluoroborane then proceeded at 55 °C for 2 h [100]. The products had a molecular weight ranging from 18,061 to 29,201 g/mol (Table 6). Among three different samples with similar molecular weights, the highest dimethomorph dispersion of 99.2% was observed using lignin modified with ethoxyethane-trifluoroborane/poly (ethylene glycol) in a molar ratio of 1/1, while dimethomorph dispersion was 93.1% using commercial lignosulfonate [100].

Lignosulfonate has also been studied as a dispersant for the dimethomorph suspension. In one study, the dispersion performance of lab-made and commercial lignosulfonates with different molecular weights and sulfonate groups were evaluated in an aqueous dimethomorph suspension (Table 7) [124]. As reported, both the lab-made and commercial lignosulfonates could act as dispersants for dimethomorph suspensions with similar results in reducing dimethomorph’s particle size. It was revealed that the lab-made lignosulfonate having a molecular mass of 16,000 g/mol contributed to the system stability through steric repulsive forces; while the commercial lignosulfonate with higher sulfonate groups provided the system stability with the electrostatic repulsive forces [124].

The effects of lignosulfonate’s molecular weight and sulfonate group content were analyzed in two other studies on the dimethomorph suspension stability (Table 7). It was revealed that the higher the polymer’s molecular weight, the greater its adsorption and thus the greater the electrostatic repulsion force between the particles, leading to the stability of dimethomorph. It was also reported that a lignosulfonate with a molecular weight higher than 30,000 g/mol was inefficient in dispersing dimethomorph due to its longer branched structure that might bridge the particles [123,126]. Lignosulfonates with similar molecular weights, but various sulfonation degrees, were also evaluated for their dispersion performance in the dimethomorph granule suspension, and the results revealed that an increase in the sulfonation degree from 1.58 to 1.81 mmol/g increased the suspending ratio by 10% [123].

In addition, the performance of sulfonated lignin with various cations of sodium, magnesium, and iron as a dispersant was evaluated and it was determined that an increase in the valence of a cation led to a slight enhancement (i.e., 4%) in the dispersion performance [123]. However, in order to increase the efficiency of lignin-based dispersants, lignin needs to undergo various chemical modifications to increase its charge density and solubility. Discovering the reactions with high efficiency and environmentally friendly features is a challenge. 

Matsushita and Yasuda produced several types of sulfonated lignin by hydroxymethylating, sulfonating, phenolating, sulfomethylating and arylsulfonating of the phenolated lignin to use as dispersants in gypsum pastes [120]. It was also claimed that the increase in molecular weight (to around 15,000 g/mol) and sulfur content (to 11.4%) enhanced the dispersibility of the gypsum paste by 70% compared to the commercial lignosulfonate [120]. 

In another work, lignosulfonate was sulfobutylated (using varied proportions of 1,4-butane sulfonate and 1,6-dibromohexane for 7 h at 70 °C) and used as a dispersant for the antifungal agent carbendazim suspensions. It was found that the modified lignosulfonates having the molecular weights of 11,850 and 13,120 g/mol with sulfonate group contents of 2.66 and 2.18 mmol/g, respectively, improved the dispersion properties of the suspension and that these samples could act as better dispersants than a commercial lignosulfonate [127].

Yang and coworkers (2008) investigated the dispersion performance of calcium lignosulfonate with different molecular weights in the range of less than 1000 g/mol to more than 30,000 g/mol in a titanium dioxide suspension (Table 7) and observed that an increase in the molecular weight from 927 g/mol to 21,646 g/mol led to an increase in the adsorption of lignosulfonate on titanium oxide particles [125]. Lignosulfonate with the molecular weight of 7621 g/mol had the highest sulfonate group content of 10.28 wt. % and showed the best dispersion performance in the dosage below 4 mg/L. System stabilization, in this case, has been pronounced to be due to the electrostatic forces between the titanium dioxide particles. Meanwhile, by increasing the modified lignosulfonate concentration, the sample with a higher molecular weight (21,646 g/mol) demonstrated a better dispersion via promoting steric hindrance.

## 5. Conclusions

Although promising results were reported for the modification and application of lignin as flocculants, adsorbents, and dispersants, the modification procedures seemed to be industrially unattractive, as they mainly applied solvent-based systems. Also, more analyses were carried out for assessing the performance of lignin-based flocculants, adsorbents, and dispersants in simulated samples than industrially produced samples. The application of these products in industrially produced samples must be examined for further development of lignin-based processes for valorizing lignin. Although the results on the importance of molecular weight and charge density of lignin-based flocculants are available in literature, the contribution of lignin in the flocculation systems should be investigated in detail. The use of lignin as a flocculant for dyes has been examined more than for other wastewater effluents. However, the volume of municipal and industrial wastes produced annually make the expansion for the potential use of lignin-based flocculants in these effluents appealing. The use of unmodified lignin as an adsorbent for dyes and heavy metals appears promising, and its simplicity in use may be attractive to industry. The uses of unmodified and modified lignin and lignosulfonate as dispersants were more practiced in the past and the reports showed a similar performance (or better) for lignin-based dispersants than for synthetic ones. Different values were reported as optimum values for molecular weight and charge density of lignin-based dispersants for altered systems. These results suggest that the properties performance relationships for lignin-based dispersants are case dependent. 

## Figures and Tables

**Table 1:** Lignin-based flocculants used in different fields.

Lignin Type	Application	Charge Density meq/g	MW (g/mol)	Modification	References
Pulping sludge containing lignin	Wastewater	N/A	510,000	Amination with triethylamine	[34]
Kraft lignin from black liquor	Dye removal	N/A	N/A	Grafting trimethyl quaternary ammonium salt	[35]
Alkali lignin	Dye removal	N/A	N/A	Grafting trimethyl quaternary ammonium salt and along with sodium alginate	[36]
Thermomechanical pulping lignin (76 wt. % lignin)	Dye removal	−3.02	6270	Nitric acid oxidation	[37]
Hydrolysis lignin	Dye removal	+1.79	2669	Dimethylamine-acetone-formaldehyde copolymer grafting, Mannich reaction	[38]
		+2.11	2762		
		+2.55	6143		
Papermaking sludge (45–50 wt. % lignin and 5–10 wt. % cellulose)	Wastewater	N/A	1000	Acrylamide graft copolymerization	[39,40]
Softwood kraft lignin	Dye removal	+1.10	21,600	Cationization with GTMAC	[11]
Softwood kraft lignin	Dye removal	−4.61	18,300	Oxidation and sulfomethylation	[41]

N/A: not available.

**Table 2:** Lignosulfonate as a flocculant in different areas.

Application as a Flocculant	Modification	Reference
Wastewater containing furfural	Radiation polymerization with olefins monomers	[42]
oil sands	No modification, but applied along with polyacrylamide-based polymers	[43]
Wastewater	Grafting with acrylamide and chitosan	[44]

NA: not available.

**Table 3:** Lignin potential in heavy metal removal.

Material	Adsorbent	Adsorption Capacity	Reference
Pb(II)	Wheat straw lignin	85%	[62]
Cr(III) + Pb, Cr(III) + Cu, Cr(III) + Zn, Cr(III) + Cd	Isolated lignin from black liquor	≥90%	[63]
Cr(VI)	Kraft lignin	33.33 mg/g	[64]
Cr(VI)	Alkali lignin	65 mg/g	[65]
Cu + Ni Cu + Cd	Kraft lignin	≥80% ≥80%	[66]
Fe(III) Mn(III)	Wheat straw lignin	100% 100%	[67]
Cu(II)	Wheat straw lignin	35 mg/g	[68]
Cd(II)	Alkynylated lignin	87.4 mg/g	[69]
Pb(II)	Aminated/esterified alkali lignin	120 mg/g	[70]
Cu(II) Pb(II)	Aminated sulfomethylated lignin	≥60% ≥60%	[68]
${\mathrm{PO}}_{4}^{2-}$	Fe-Aminated lignin complex	≥90%	[71]
Al(III) Co(II) La(III)	Crosslinked lignocatechol	80% 100% 100%	[72]
${\mathrm{Ag}}^{+}$	Lignin-polyaniline	1556.8 mg/g	[15]
Cu(II) Cd(II)	Lignin-melamine formaldehyde	73.2 mg/g 142.3 mg/g	[73]

**Table 4:** Lignin as an adsorbent for dye removal.

Material	Adsorbent	Adsorption Capacity	Reference
Brilliant Red HE-3B dye	Wheat straw lignin	10.17 mg/g	[50]
Methylene Blue dye	Esterified Lignin	31.23 mg/g	[74]
Brilliant Red 2BE dye	Etherified lignin-${\mathrm{Fe}}^{3+}$ complex	73.6 mg/g	[75]
Anthraquinonic dye	Chitosan-alkali lignin complex	≥90%	[76]
Procion Blue dye	Carboxymethylated lignin-${\mathrm{Al}}^{3+}$ complex Carboxymethylated lignin-${\mathrm{Mn}}^{2+}$ complex	73.52 mg/g 55.16 mg/g	[77]
Methylene Blue	Reticulated formic lignin	34.12 mg/g	[50]

**Table 5:** Lignin as an adsorbent in other chemicals.

Material	Adsorbent	Adsorption Capacity	Reference
TNT	Chlorinated aminated lignin	55.7 mg/g	[79]
2-nitrophenol	Hydrolysis lignin	1.8 mg/g	[81]
Bisphenol	Black liquor isolated lignin	237.07 mg/g	[82]
Metamitron, metribuzin pesticide	rot-wood lignin	53% 62%	[83]
Dazomet/tiram pesticide	Indulin kraft lignin	38–40%	[84]
Hexazinone pesticide	Indulin kraft lignin	47%	[85]
Au(II)	Crosslinked lignophenol	≥30%	[86]
Au(III)	HCl mediated kraft lignin	100%	[77]
Au(III) Pd(II)	Aminated lignin	100% 80%	[86]

**Table 6:** Proposed lignin-based dispersants for various fields.

Lignin Type	Application	Charge Density (meq/g)	Molecular Weight (g/mol)	Modification	References
Hardwood kraft lignin	Cement admixture	−1.60	53,360	Sulfomethylation	[94]
Softwood kraft lignin	Stellar clay, cement, calcium carbonate and titanium dioxide	N/A	N/A	Ozone oxidation	[95]
Softwood kraft lignin	Kaolin suspension	−2.2	14,825	Oxidation	[96]
Lignin N/A	Carbon nanotubes nanofluids	N/A	N/A	As is	[97]
Straw alkali lignin	Dispersant for graphite suspension	N/A	N/A	Carboxymethylation	[98]
Softwood kraft lignin	Dispersant for cement admixture	−3.8	18,299	Oxidation and sulfomethylation	[44]
Hardwood lignin	Kaolin suspension	(−)1.2–3.62	26,700–83,543	Oxidation	[99]
Hardwood kraft lignin	Kaolin suspension	1.80	29,960	Carboxymethylation	[94]
Kraft lignin	Dimethomorph suspension	N/A	18,061–29,201	Grafting poly(ethylene glycol) functionalized with epichlorohydrin using BF_3_-Et_2_O	[100]
Wheat straw kraft lignin	Cement admixture	N/A	25,700	Sulfonation	[101]
Wheat straw alkali lignin	Cement admixture	N/A	9688	Hydroxymethylation and sulfonation	[102]
Pinewood alkali lignin	Dye suspension	N/A	11,020	Hydroxypropylation and sulfonation	[103]
Spruce alkali lignin	Multiwalled carbon nanotubes	N/A	14,000	As is	[104]
Softwood kraft lignin	Multiwalled carbon nanotubes	N/A	6500–7000 and 34,000–36,000	Fractionization	[105]
Wheat straw alkali lignin	TiO_2_ slurry	N/A	17,400–35,700	Sulfomethylation, horseradish peroxide utilization	[106]
Esparto grass lignin	Cement admixture	N/A	10,000	Sulfonation	[107]
Acid precipitated lignin	Cement admixture	N/A	N/A	Sulfonation	[108]

N/A: not available.

**Table 7:** Lignosulfonates as dispersants in various applications.

Lignosulfonate Type	Application	Charge Density (meq/g)	Molecular Weight (g/mol)	Modification	Reference
NA	Electroceramic suspensions	−0.061 ± 0.002 C/m^2^	37,000	No modification	[119]
NA	Gypsum paste	NA	9000–62,000	Hydroxymethylation, sulfonation, phenolation, sulfomethylation, arylsulfonation	[120]
NA	Coal-water slurry	NA	2000-17,000	No modification	[59]
Sodium lignosulfonate	Coal-water slurry	NA	less than 5000 to more than 50,000	No modification	[121]
Calcium lignosulfonate	Titanium dioxide suspension	NA	less than 1000—more than 30,000	No modification	[122]
Calcium lignosulfonate	Cement admixture	NA	NA	Oxidation, sulfomethylation, hydroxymethylation	[118]
NA	Dimethomorph suspension	NA	Less than 1000—more than 30,000	No modification	[123]
Hardwood lignosulfonate	Cement admixture	NA	NA	No modification	[120]
NA	Dimethomorph suspension	NA	4800–160,000	No modification	[123]
Sodium lignosulfonate	Ceramic suspension	NA	13,000	No modification	[124]
Sodium lignosulfonate	Concrete admixture	NA	2378 and 23,650	Oxidation, sulfomethylation	[115]
NA	Dye suspension	NA	9010–17,307	No modification	[125]
NA	Dimethomorph suspension	NA	9600–35,500	Oxidation, sulfonation	[126]
NA	Carbendazim suspension	NA	1900–13,120	Sulfobutylation	[127]
NA	Coal-water slurry	NA	13,100–251,000	Alkyl chain coupling polymerization	[128]
Calcium, magnesium, sodium, potassium lignosulfonate	Cement admixture	NA	NA	No modification	[117]

NA: not available.
